# Nausea and Vomiting as the Reasons for Encounter in General Practice

**DOI:** 10.4021/jocmr410w

**Published:** 2011-02-12

**Authors:** Thomas Frese, Steffi Klauss, Kristin Herrmann, Hagen Sandholzer

**Affiliations:** a Department of Primary Care, Leipzig Medical School, Leipzig, Germany

## Abstract

**Background:**

The present study aimed to explore the consultation prevalence, differential diagnoses, and management of patients presenting with nausea or vomiting to their family doctors.

**Methods:**

Cross-sectional data were collected from randomly selected patients during the SESAM 2 study (October 1, 1999 to September 30, 2000). We contacted 2510 doctors; 270 (10.8%) of them participated in the study. Data were collected from randomly selected patients previously known to the general practitioner. Unpublished but publicly available data from the Dutch Transition Project were also analysed.

**Results:**

One hundred and sixty-nine of the total 8874 patients consulted their general practitioner for nausea/vomiting; 97 (57.4%) were female and 72 (42.6%) were male. Most patients suffering from nausea or vomiting in general practice were aged between 15 and 64 years. Nearly all patients were given a physical examination. Most diagnoses were made without further investigation, additional diagnostic procedures were found to be necessary in only 7 patients. Drugs were prescribed as the most frequent form of medical treatment, in 76.3% of cases. Non-infectious gastroenteritis or colitis was the most frequent diagnosis. Nausea or vomiting was associated with diarrhoea, fever, and abdominal pain. Headache, general weakness, and epigastric pain were also statistically significantly associated with nausea or vomiting.

**Conclusions:**

Many disorders cause nausea or vomiting. Although most of the patients were diagnosed with non-infectious gastroenteritis or colitis, the general practitioner also has to bear in mind that nausea and vomiting may be alarm symptoms. Medication was prescribed in most of the cases and there were only a few referrals to a specialist or hospital. Life-threatening disorders (appendicitis, bowel obstruction/ileus) were found in a few cases presenting with nausea or vomiting.

**Keywords:**

Nausea; Vomiting; General practice; Primary care

## Introduction

A 60-year-old patient comes to your surgery because of nausea. Is this a simple case or is it due to myocardial infarction, intracerebral bleeding, stroke, or gastric ulcer? Nausea and vomiting, both separately and together, are common complaints in general practice [1-3]. Vomiting is a protective reflex intended to remove potential harmful or toxic substances from the body [4, 5] and to moderate pressure in hollow organs, i.e. the digestive tract [5]. The vomiting centre in the medulla oblongata and the chemoreceptor trigger zone (area postrema) located at the base of the fourth ventricle [5] control vomiting. Stimulating these centres triggers a series of coordinated motor events to induce vomiting [5]. This is accompanied by an increase in salivation [4] and sweating, and a decrease in gastric tone. Nausea is the unpleasant feeling of being about to vomit; it is often accompanied by increased salivation [4]. This sensation seems to be caused by elevated muscle tone in the stomach [5]. In determining the reason for nausea or vomiting, a first consideration is whether the symptoms are acute or chronic. Symptoms lasting one month or longer are deemed chronic [4]. Nausea or vomiting constitutes an appreciable reason for encounter. An Australian study showed that 1.6% (1.5 million encounters) of the consultations in a primary care setting per year were for nausea or vomiting [6]. Although nausea and vomiting are common reasons for encounter in general practice [2] and the illness is often self-limiting [4], vomiting may be an alarm symptom [7, 8]; so it is important to detect complications and eliminate acute emergencies [4]. But, there are very few current data on these two symptoms. The present study aimed to explore the consultation prevalence, differential diagnoses, and management of patients presenting with nausea or vomiting to their family doctors.

## Methods

The Saxon Society of General Medicine (SGAM) contacted all 2510 general practitioners in Saxony: 270 of them agreed to participate and 209 cooperated fully. Cross-sectional data were collected from October 1, 1999 to September 30, 2000. Case recording was performed on one day each week (Monday to Friday; either morning or afternoon surgery hours), chosen at random. We collected data for one in ten, and did not record the same patient twice. We did not take home visits into account. A total of 8877 patients were included. We used a standardized data collection form developed by general practitioners (Leipzig Medical School and Saxon Society of General Medicine). The form was tested and evaluated during a pilot trial (SESAM 1). It was found to be relevant, cost-effective, and time-efficient. Each patient's reason for consultation, symptoms, diagnostic process, recent diagnoses and general morbidity were documented, as well as any therapeutic measures. As far as possible, data were recorded verbatim (according to the study protocol): either as reported by the patient (e.g. reason for encounter) or in the words of the physician (e.g. chronic diagnoses). Because of the random selection, the information was documented in a reasonably short time. Only fully completed forms were taken into account. The International Classification of Primary Care (ICPC), 1987, was used to code the reasons for encounter [9]. Unpublished but publicly available data from the Transition Project [10] were also analysed. Data analysis was carried out using the Statistical Packages for Social Sciences (SPSS 15.0 Inc., Chicago, USA).

## Results

We documented a total of 8877 consultations. The number of cases reported from each doctor's surgery ranged from 23 to 54. Five thousand fifty (56.9%) female and 3824 (43.1%) male patients were included; gender was not reported in 3 cases. Ages ranged from 2 to 102 years (mean 51.2 years, SD 20.86, median 55 years). One hundred and sixty-nine patients, 97 (57.4%) of them female, reported nausea or vomiting. As shown in Table 1, nausea or vomiting had higher consultation prevalences in young patients. The consultation prevalence showed no gender-related differences. There were no seasonal changes in the incidence.

**Table 1:** Patients Distribution (pd) and Consultation Prevalence (cp) of Nausea/Vomiting in Different Age Groups in the German SESAM 2 Study and the Dutch Transition Project regarding New or Previously Known Nausea/Vomiting

Age (years)	SESAM 2 Study (n = 169, total n = 8877)		Transition Project (new) (n = 3215, total n = 219596)		Transition Project (known) (n = 1052, total n = 219596)	
	pd [%]	cp [%]	pd [%]	cp [%]	pd [%]	cp [%]
0 to 4	1.8	2.7	19.5	4.59	9.4	0.72
5 to 14	8.3	4.9	13.4	1.71	7.3	0.31
15 to 24	32.0	6.1	13.8	1.69	11.1	0.45
25 to 44	32.0	3.0	25.7	1.16	29.7	0.44
45 to 64	19.5	1.1	13.9	0.97	18.7	0.43
65 to 74	3.0	0.3	7.8	1.24	12.3	0.64
> 75	3.6	0.5	5.8	1.1	11.5	0.71

A few accompanying symptoms were significantly associated with nausea or vomiting: people complaining of nausea or vomiting in the SESAM study suffered significantly more often from colic, abdominal pain, diarrhoea and headache (*p* < 0.001 for each). Other associated symptoms were fever (*p* = 0.003), heartburn (*p* = 0.004), haematemesis (*p* = 0.019) and weakness (*p* = 0.024) as can be seen in Table 2.

**Table 2:** Symptoms Significantly Associated With Nausea/Vomiting (SESAM 2 study)

Diagnosis	Nausea/Vomiting		Without Nausea/Vomiting		*p* value (Fisher)
	absolute	%	absolute	%	
Diarrhoea	65	38.5	102	1.2	0.000
Colic	21	12.4	140	1.6	0.000
Fever	16	9.5	357	4.1	0.003
Headache	13	7.7	168	1.9	0.000
Abdominal pain	12	7.1	98	1.1	0.000
Feeling of weakness	8	4.7	176	2.0	0.024
Heartburn	4	2.4	30	0.3	0.004
Haematemesis	1	0.6	-	-	0.019

Nearly all patients were given a physical examination (94.1%). Most diagnoses (95.9%) were made without further investigation; only seven patients underwent additional diagnostic procedures (e.g. ultrasound scanning or scatoscopy). Table 3 shows that the most frequent types of medical treatment were the prescription of drugs (76.3%) and medical advice (20.2%), reflecting the diagnoses of acute infections or diseases not otherwise specified.

**Table 3:** Diagnostic and therapeutic Procedures [%] in the German SESAM 2 Study and the Dutch Transition Project regarding New or Previously Known Nausea/Vomiting

Procedure	SESAM 2 study (n = 169)	Transition Project (new) (n = 3215)	Transition Project (known) (n = 1052)
Physical examination	94.1	82.7	69.6
Drug prescription	76.3	46.4	47.1
Follow-up consultation	64.5	-	-
Incapacity to work	53.8	-	-
Medical advice	20.2	43.4	34.3
Laboratory investigations	16.0	14.5	15.4
Long-term care until now	14.8	-	-
Other therapy	10.7	0.03	-
Other diagnostics	5.3	0.34	1.5
Hospitalisation	5.3	2.7 (sc)	9.6 (sc)
History	4.7	-	-
Referral	4.7	0.5 (pc)	0.7 (pc)
ECG	2.4	-	-

pc: primary care; sc: specialist care

There were no serious acute conditions except appendicitis and bowel obstruction/ileus. In addition, a specific early complication of trauma, surgical intervention or medical treatment (e.g. wound infection or complication after anaesthesia) was made in four cases (2.4%). The diagnoses are summarised in Table 4.

**Table 4:** Prevalence [%] of Diagnoses in Patients With Nausea or Vomiting in the Different Studies

Diagnosis	SESAM 2 Study (n = 169)	Transition Project (known (n = 3215)	Transition Project (known) (n = 1052)
Other disease of the digestive system (non-infectious gastroenteritis, colitis)	28.4	1.0	0.6
Stomach upset	21.3	14.2	10.0
Infectious diarrhoea/dysentery	12.4	0.4	1.1
URI (head cold)	8.9	0.8	2.1
Migraine	4.7	1.5	1.1
Presumed gastrointestinal infection	3.0	7.2	21.4
Appendicitis	3.0	0.3	0.7
Nausea/vomiting	2.4	-	-
Adverse drug effect	1.8	2.6	4.1
Cirrhosis/other liver disease	1.8	0.8	0.1
Chronic alcohol abuse	1.8	0.4	0.1
Fever	1.2	0.1	0.1
Acute bronchitis/bronchiolitis	1.2	1.3	1.2
Disease of oesophagus	1.2	2.4	0.7
Pyelonephritis/acute pyelitis	1.2	0.3	0.2
Other peptic ulcers	1.2	0.95	0.3
Other viral diseases NOS	0.6	1.3	5.2
Cholecystitis/cholelithiasis	0.6	1.8	0.8
Nausea/vomiting of pregnancy	0.6	0.95	0.7
Anorexia nervosa	0.6	0.3	0.1
Acute stress	0.6	0.6	0.6
Malignancy (digestive system)	-	0.8	0.3
Heartburn	-	0.5	0.2
Duodenal ulcer	-	2.2	0.2
Inguinal hernia	-	-	0.1
Nausea	-	10.1	12.4
Vomiting	-	5.1	8.1
Vertigo syndromes	-	1.4	1.7
General weakness/tiredness	-	0.8	1.6
Constipation	-	1.7	1.6
Irritable bowel syndrome	-	6.1	1.1
Concussion/head injuries	-	0.2	0.8
Haematemesis/vomiting blood	-	-	0.06

A total of 219,596 patients were examined in the Transition Project; 114,495 (52.1%) of them were female. Among the total patients, 3215 patients declared nausea or vomiting as the reason for consultation, 2003 (62.3%) of these patients were female; 1774 patients consulted for nausea and 1541 for vomiting, 100 patients complained of both. The age distribution of the patients is given in Table 1. Patients presenting with nausea or vomiting often also suffered from diarrhoea (11.7%), fever (11.7%), and abdominal pain (other localized; 5.9%). Headache (5.4%), general weakness or tiredness (4.8%) and epigastric pain (4.7%) were also statistically significantly associated with nausea or vomiting. Moreover, the two symptoms were often accompanied by generalised abdominal pain/cramps (4.6%), vertigo or dizziness (3.7%), cough (3.5%) and heartburn (1%). Most patients were given a physical examination (Table 3). Medication was prescribed or injected in 46.4%. Health education or medical advice was regularly given. Further diagnostic investigation was not necessary in most of the cases (Table 3). A referral to other doctors or a hospital was made in 87 cases (2.7%). Forty-five (51.7%) patients were sent as emergency admissions and the remainder (n = 42, 48.3%) were referred to specialists less urgently. Fifteen patients were referred to an emergency surgeon, 11 (12.6%) to a paediatrician (non-emergency), 11 (12.6%) to a general surgeon (non-emergency) and 9 (10.3%) to an emergency paediatrician. The remaining patients (n = 41) were referred to other specialists, including specialists in general medicine (emergency and non-emergency) and emergency cardiologists. Most patients consulting their general practitioner for nausea or vomiting were coded as having a presumed gastrointestinal infection (21.4%), followed by nausea per se (12.4%). Stomach upset (10.0%) and vomiting per se (8.1%) were found in third and fourth places. A diagnosis of viral infection (not otherwise specified) was made in 168 cases (5.2%), ranking fifth.

## Discussion

The SESAM 2 study supplies impartial and independent data collected within a routine primary care setting [11]. Nausea and vomiting are common complaints for a general practitioner to deal with [1-3]. Nevertheless, there are hardly any surveys on these reasons for encounter. Recent data have been ascertained in just one study [6]. The consultation prevalence of nausea or vomiting in the studies considered varies within a small range from about 1.5% in the Transition Project to 1.9% in the SESAM 2 study. The BEACH study (prevalence 1.6%) showed no difference in gender-specific presentation rates [6]. This is confirmed by our investigation. Women accounted for 57% of the cases with nausea or vomiting, but they also represent 56% of all patients. However, in the Transition Project, the male to female ratio was about 1.7:1. We found the highest consultation prevalence of nausea or vomiting in children and adolescents (Table 1). The BEACH study found vomiting more frequently in children younger than 15 years [6]. Nausea was recognized more often in patients aged 15 - 24 years [6]. The Transition Project shows that most patients consulting for nausea or vomiting are 25 to 44 years old (Table 1). As previously mentioned, vomiting was found most frequently in children aged 0 - 15 years in the Transition Project, as well as in the BEACH study. It should be regarded that the age distribution of the patients is different among the three studies (Table 1). In Germany, children aged 0 - 4 years are mostly treated by paediatricians, which may explain the low overall percentage of this age group in our patients compared with the Transition Project, and thereby the Dutch healthcare system (Table 1). The study performed by Kuehlein et al [12] illustrates the top 20 reasons for encounter according to gender and age. In the age group 0 - 44 years, there was an incidence of 1.2% - 1.7% for nausea and 1.2% - 6.7% for vomiting. Gender-related differences in the incidence of nausea or vomiting were found in children and adolescents. However, the findings in the different studies are not consistent and may be assumed to have no relevance.

In both the SESAM 2 study and the Transition Project, nearly all patients presenting with nausea or vomiting had a medical examination or health evaluation. Drug prescription was the most frequent therapeutic procedure in both studies. Patients with nausea or vomiting were seldom referred to a specialist, and hospitalisation was necessary in only a few cases. General practitioners can deal adequately with nausea or vomiting as the reason for encounter. In the vast majority of cases, symptomatic treatment (drugs, diet, and time off work) is all that is necessary. Some integrative therapies have also shown benefit in the management of these symptoms, specifically for nausea induced by pregnancy, chemotherapy, and surgery [2]. In contrast to encounters for other reasons, e.g. fatigue [13], there is no watchful waiting strategy and the diagnosis is made at the initial consultation [14].

Gastroenteritis was the most frequent diagnosis made by general practitioners in the studies considered (Table 4). This diagnosis was made in 37 per 100 encounters in the BEACH study [6]. As stated by other authors, gastrointestinal infections and food poisoning are the most common causes of acute nausea and vomiting [15]. In children, viral gastroenteritis is the most common cause and management of hydration is vital [4]. But, as suggested by Britt et al [6], upper respiratory tract infections and otitis media should also be considered as underlying causes in children and young people [6]. Regular review in the early phases of an undifferentiated vomiting illness will ensure that dehydration does not occur. Chronic regurgitation, gastro-oesophageal reflux, and food allergy may cause nausea or vomiting in infancy [4]. This was not confirmed by our findings, but Britt et al did not rate these causes as common [6]. Functional vomiting is rare: it presents with more frequent vomiting episodes and should be distinguished from rumination [1]. The possible diagnoses in patients presenting with nausea or vomiting differ with age [6]. Most of the recent literature seems to deal with perioperative, chemotherapy-, and radiotherapy-induced nausea and vomiting. These palliative situations seem not to be common problems in a routine primary care setting. Adverse effects of medical treatment had to be managed in 3% of the patients complaining about nausea or vomiting, but they were seen more often in patients over the age of 65 [6]. This may be due to the increased prescription of drugs for older patients. But the symptoms of nausea and vomiting reported by patients also remained undiagnosed, being described merely as nausea or vomiting, in one out of ten encounters [6]. In contrast to the SESAM 2 study, a simple diagnosis of nausea or vomiting was also made in a number of encounters in the Transition Project. This may be due to differences in the study designs. The Transition Project covers a broader spectrum of diagnoses (Table 4) than we found in our study, as there is a much bigger sample. General practitioners participating in the SESAM 2 study mostly made a diagnosis of non-infectious gastroenteritis and colitis, while general practitioners taking part in the Transition Project often made a diagnosis of presumed gastrointestinal infection, as there may have been more testing. Differences in the frequency of diagnoses may also be explained by the fact that Dutch general practitioners often coded for nausea or vomiting as a symptom and not as diagnosis per se (in contrast to the general practitioners in the SESAM 2 study). Unlike the Transition Project, we did not find gastrointestinal malignancies, haematemesis. This indicates that only a few conditions were serious. Problems and the necessity for hospitalisation resulted from appendicitis, bowel obstruction/ileus or dehydration due to persistent vomiting. The SESAM 2 data include no follow-up; they represent initial consultations and not episodes of care. This might mean that not all serious conditions were diagnosed and some "black swans"(note 1) [16] may have been overlooked. However, it should be remembered that the SESAM 2 study did not take home visits or urgent out-of-hours consultations into account. Even though nausea is a common symptom of, for example, acute myocardial infarction [17, 18], it can be seen that, due to a low pre test probability, the patient who presents with nausea as the *only* symptom of his myocardial infarction is a "zebra" (note 2) in general practice.

## Conclusion

Nausea and vomiting are common complaints presenting in general practice. Many disorders cause nausea or vomiting or are accompanied by these symptoms. The most common diagnosis was gastroenteritis, but in a few cases the presenting complaint remained undiagnosed. Most of the patients did not require referral to a specialist or hospital, but a number of conditions presenting with nausea or vomiting may be serious. Although further diagnostic procedures may be necessary to avoid preventable harm, general practitioners should not be afraid of "zebras".

## Notes

1. A "black swan" is a term coined for a highly improbable event with three principal characteristics: it is unpredictable; it carries a massive impact; and, after the fact, we concoct an explanation that makes it appear less random, and more predictable, than it was.

2. Zebra is a medical slang term for an obscure and unlikely diagnosis from ordinary symptoms. It derives from the aphorism "When you hear hoofbeats, think horses, not zebras.".
